# Resection versus preservation of the middle turbinate in surgery for chronic rhinosinusitis with nasal polyposis: a randomized controlled trial

**DOI:** 10.1186/s40463-018-0313-8

**Published:** 2018-11-08

**Authors:** Marc-Antoine Hudon, Erin D. Wright, Etienne Fortin-Pellerin, Marie Bussieres

**Affiliations:** 1 Université de Sherbrooke, 580 Rue Bowen S, Sherbrooke, QC J1G 2E8 Canada; 2 University of Alberta, Room 1E4, W.C.M. Health Sciences Centre, 8440-112 Street, Edmonton, AB T6G 2B7 Canada; 3 Université de Sherbrooke, 3001, 12e avenue Nord, Sherbrooke, QC J1H 5N4 Canada

**Keywords:** Chronic rhinosinusitis, Nasal polyposis, Endoscopic sinus surgery, Middle turbinate

## Abstract

**Background:**

Chronic rhinosinusitis (CRS) affects up to 16% of the population. When medical treatment fails, endoscopic sinus surgery (ESS) is considered. The value of resecting the middle turbinate to optimize surgical outcomes has been hypothesized but remains controversial and unproven. Whether the middle turbinate should be left in place or resected is controversial. Our objective is to determine if middle turbinectomy improves objective surgical outcomes after ESS.

**Methods:**

Sixteen patients (15 men, 15 primary surgery) undergoing bilateral complete ESS for CRS with nasal polyposis were recruited. Nasal cavities were randomized so that middle turbinectomy was performed on one side while the middle turbinate was preserved on the other. Each participant acted as their own control. Nasal cavities were compared using Perioperative Sinus Endoscopy (POSE) and Lund-Kennedy (LKES) scores pre-operatively, and at 1, 3 and 6 months after ESS. Results were analyzed using Wilcoxon signed-rank test.

**Results:**

Pre-operatively, the POSE (12.4 ± 2.9 vs 12.8 ± 2.6, *p* = 0.33, for the preserved side and the resected side, respectively) and LKES (5.0 ± 1.0 vs 4.8 ± 1.2, p = 0.33) scores were similar between sides. During follow up, resection was associated with more crusting at 1 month following ESS (1.0 ± 0.7 vs 0.4 ± 0.6, *p* = 0.02). There was a small, but statistically significant, difference between the nasal cavities at 3 months, where the resected side showed better endoscopic appearance (2.0 ± 2.2 vs 3.4 ± 2.8, *p* = 0.01). No difference was found at 6 months. Frontal sinus scores were similar between sides at 6 months (0.7 ± 0.5 vs 0.7 ± 0.5, *p* = 1.00).

**Conclusion:**

Our results show no sustained objective endoscopic benefit of routine middle turbinectomy, at least within the first six postoperative months, in patients undergoing primary ESS for CRS with polyposis.

**Trial registration:**

NCT, NCT02855931. Registered 16 August 2016.

## Background

Chronic rhinosinusitis (CRS) is a common disease affecting up to 16% of the population [1]. Medical expenses related to CRS reach more than 60 billion dollars per year in the United States alone [2], with an additional 13 billion dollars per year [3] in loss of productivity.

Medical treatments, consisting of nasal saline irrigations, topical and systemic corticosteroids, are first offered to the patients. If symptoms persist, endoscopic sinus surgery (ESS) can be recommended [4]. The surgery has multiple goals such as removal of gross disease, marsupialization of sinus cavities, clearance of inspissated secretions and improved access of post-operative topical medical therapies [5]. The role of middle turbinectomy in ESS remains controversial. Traditionally, this structure has been preserved in order to maintain the integrity of the nasal cavity as much as possible. Removal of the middle turbinate was deemed to be hazardous, with some authors advocating it could lead to increased risk of iatrogenic frontal sinusitis [6, 7]. This, however, has been refuted by Saidi et al. [8]. Removal of the middle turbinate might also increase the difficulty of revision surgeries, since it is an important anatomic landmark [6]. On the other hand, some authors have suggested resection could allow for more efficient nasal irrigations and topical corticosteroids owing to improved access, potentially leading to reduced polyp recurrence in the long term [5]. Retrospective studies have demonstrated longer time lapse before revision surgery [9], better endoscopic scores [10] and less synechiae with resection of the middle turbinate [11]. Unfortunately, there is very limited prospective data specifically looking at this issue [12]. More importantly, available studies were not randomised, leaving the decision as to whether to resect or preserve the turbinate at the surgeon’s discretion, thus introducing a significant bias [10].

Our goal was to prospectively evaluate the role of middle turbinectomy on endoscopic outcomes of patients undergoing ESS for CRS with polyposis. Our hypothesis was that resection of the middle turbinate would improve sinonasal cavities appearance, as assessed by the POSE and the LKES scores.

## Methods

A randomized controlled trial was conducted on patients undergoing bilateral complete ESS for CRS with nasal polyposis in a rhinology tertiary care center (Centre Hospitalier de l’Université de Sherbrooke, Sherbrooke, Canada). Ethics approval was obtained from the institutional ethics board (Comité d’éthique de la recherche en santé chez l’humain du CIUSSS de l’Estrie – CHUS). The protocol was registered prior to patient enrollment (clinicaltrials.gov - NCT02855931).

Sample size calculation was based on a study using a similar design [13]. Thirty-two nasal cavities were required to detect a difference of 3.5 points in POSE score (alpha 0.05, 80% power). A difference of 3.5 points in the POSE score is considered clinically significant [14].

Patients were recruited if they were above 18 years of age with a diagnosis of CRS with nasal polyposis. Patients undergoing both primary and revision surgeries were included. Patients were excluded if they had a diagnosis of allergic fungal rhinosinusitis, if the middle turbinate had been resected during a previous surgery, or if they were pregnant. General data on age, sex, asthma, smoking, airborne allergies and postoperative epistaxis were collected. Prior to the surgery, the Lund-Mackay radiologic scoring system [15] was used to assess the degree of opacification of the sinus cavities, a higher score correlating with more severe disease (six regions evaluated on each side, scored 0–2, total maximum score of 12). Informed consent was obtained prior to surgery, which consisted of bilateral polypectomy, maxillary antrostomy, sphenoethmoidectomy and frontal sinusotomy (Draf 2a surgery). Each participant had the middle turbinate resected completely on one side and preserved on the other and were consented accordingly. Participants acted as their own control. Treatment allocation for choice of nasal cavity was done using computer-based block randomization, irrespective of the appearance of the middle turbinate (ex. polypoid, destabilized or with paradoxical curvature). At the end of surgery, Nasopore (Stryker Canada, Hamilton, Canada) impregnated with triamcinolone (40 mg/mL) was inserted in each ethmoid cavity. Patients were given a 7-day course of antibiotics and gentle saline irrigations. As per our routine postoperative protocol, they were seen 1 week after surgery for debridement of their sinonasal cavities and then were instructed in using budesonide nasal irrigations twice daily on a long-term basis (2 ml of 0.5 mg/ml budesonide in 240 ml of saline water). The study was single-blinded as participants were unaware of which side was resected. The investigators could not be blinded during follow-up due to the nature of the intervention.

Patients were evaluated at 1, 3 and 6 months post-operatively by the main investigator. Two clinically validated endoscopic scores were used to assess the nasal cavities. The Lund-Kennedy Endoscopic Scoring system (LKES) was used to evaluate the presence of polyps, edema, secretions, synechia and crusting in the sinonasal cavities (5 items scored 0–2 for a total maximal score of 10 on each side) [16]. The Peri-Operative Sinus Endoscopy (POSE) score adds information on the appearance of different parts of the sinonasal cavities. The middle turbinated is examined for synechia, lateralization or narrowing of the middle meatus. The maxillary, frontal and sphenoid sinuses are scored separately with regards to their healthiness or the presence and severity of mucosal edema and secretions (thin or mucoid vs purulent or mucinous). The ethmoid cavity is further inspected for signs of crusting, polypoid changes or frank polyposis. There are10 items scored 0–2 for a maximal score of 20 on each side [17]. Higher values indicate worse disease in both scores.

Statistical analysis was performed with SPSS 19 (IBM, Chicago, IL, USA). A non-parametric statistical approach (Wilcoxon signed-rank test) was chosen due to the relatively small number of patients. However, data distribution was qualitatively fairly normal and thus the authors have decided to present the results as average ± standard deviation (SD) for ease of understanding.

## Results

Sixteen patients (47.5 ± 13.6 years old) were recruited between April 2016 and July 2017. Our cohort mostly consisted of middle-aged men who had primary surgery (Table 1). None presented post-operative epistaxis.

**Table 1:** Patient characteristics

	Number of participants n (%)	*p*-value
Age (mean ± SD)	48.5 ± 13.6 years	
Sex		
Male	15 (94)	
Female	1 (6)	
Surgery type		
Primary	15 (94)	
Revision	1 (6)	
Asthma	4 (25)	
Aspirin-exacerbated respiratory disorder	1 (6)	
Environmental allergies	4 (25)	
Smoking status		
Yes	1 (6)	
No	15 (94)	
Baseline radiologic Lund-MacKay score		
Resected side (mean ± SD)	8.8 ± 2.1	*p* = 0.24
Preserved side (mean ± SD)	8.8 ± 2.5	

**Fig. 1:**
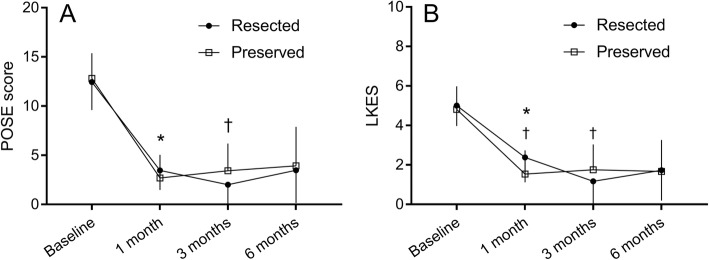
Sinonasal endoscopic outcomes after surgery. Trends for POSE (**a**) and LKES (**b**) scores over time. * First timepoint where scores within the same groups are statistically different from baseline. † Significant difference between groups at the indicated timepoint. POSE: Peri-Operative Sinus Endoscopy, LKES: Lund-Kennedy Endoscopic Score

At baseline, POSE and LKES scores were very similar between the 2 nasal cavities (12.4 ± 2.9 vs 12.8 ± 2.6, *p* = 0.33 and 5.0 ± 1.0 vs 4.8 ± 1.2, *p* = 0.33, for the side allocated to resection and the side allocated to preservation, respectively *n* = 16). Compared to pre-operative score, there was a significant improvement in the POSE score postoperatively on both sides which persisted throughout the 6-month follow-up period (*p* < 0.001) (Fig. 1a). The differences between the 2 sides at each time point, however, were minimal. Three months after ESS, there was a statistically significant but clinically limited difference favoring the resected side (2.0 ± 2.2 vs 3.4 ± 2.8, *p* = 0.01, *n* = 12) that was not present at 1 month (3.5 ± 2.0 vs 2.7 ± 2.4, *p* = 0.06, *n* = 13) and did not persist at 6 months (3.5 ± 3.3 vs 3.9 ± 4.0, *p* = 0.76, *n* = 15). The LKES scores globally followed the same trend as the POSE scores, showing better endoscopic appearance for both sinus cavities after surgery as compared to pre-operatively (*p* < 0.001). LKES values were higher (worse) at one month on the resected side (2.4 ± 1.3 vs 1.5 ± 1.2, *p* = 0.03, *n* = 13) but lower (better) at 3 months (1.2 ± 1.5 vs 1.8 ± 1.3, *p* = 0.05, *n* = 12). Scores were the same in both groups at 6 months (1.7 ± 1.5 vs 1.7 ± 1.6, *p* = 0.83, n = 15) (Fig. 1b).

**Fig. 2:**
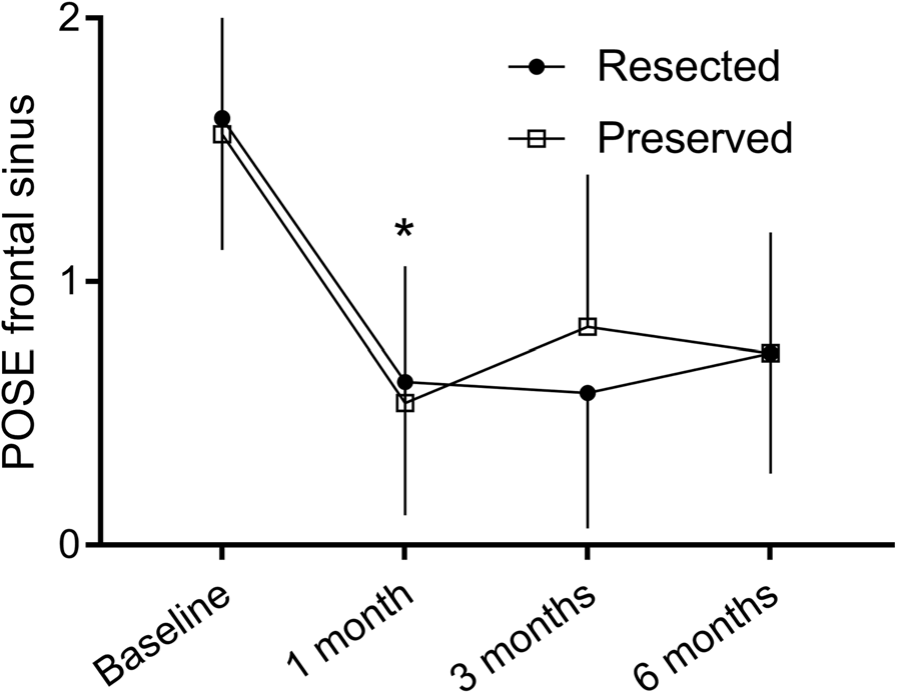
Endoscopic outcomes of the frontal sinus/recess. Trends for frontal sinus/recess subcategory of the POSE score over time. * First timepoint where scores within the same groups are statistically different from baseline. POSE: Peri-Operative Sinus Endoscopy

Analysis of individual POSE scores’ criteria showed significantly more crusting on the resected side at one month (1.0 ± 0.7 versus 0.4 ± 0.6, *p* = 0.003), but not afterwards. Synechia were seen in 3 patients on the preserved side at 6 months after surgery compared to none on the resected side. The frontal recess and sinus scores were better at every follow up visit after ESS compared to the baseline data on both sides (*p* = 0.001) (Fig. 2). Still in the frontal recess and sinus region, resected and preserved sides were similar at 1 (0.6 ± 0.5 vs 0.5 ± 0.5, *p* = 0.32, preserved and resected side, respectively), 3 (0.6 ± 0.5 vs 0.8 ± 0.6, *p* = 0.18) and 6 (0.7 ± 0.5 vs 0.7 ± 0.5, *p* = 1.00) months after surgery.

## Discussion

The role of middle turbinectomy during ESS is a matter of debate for the treatment of CRS. Some authors have found advantages to resection, as discussed earlier. Unfortunately, most of the available evidence comes from retrospective studies and were not randomized, thus introducing a significant bias [9, 18, 19]. To our knowledge, this is the first prospective randomized controlled trial to evaluate the potential of middle turbinectomy in improving outcomes after ESS for CRS with polyposis. Although there were transient differences between the 2 approaches, we found no objective persistent advantage of middle turbinectomy in the surgical treatment of CRS patients.

We found a statistically significant difference in POSE scores in favour of middle turbinate resection 3 months after surgery. The amplitude of this difference, however, was small enough to be arguably of limited clinical relevance. Moreover, it did not persist at 6 months. This was an unexpected finding. Since there is evidence of better access of topical medication in a completely marsupialized sinus cavity [20], we were expecting a sustained improvement on the side of middle turbinate resection after ESS. More specifically, we thought the improved access of postoperative medication would make a difference in the region of the frontal recess where early recurrence of polyposis is usually seen. Even though we found no significant added benefit of resection, it is noteworthy that there was no adverse effect of resection, showing the middle turbinate can be removed safely if deemed clinically indicated. Despite our negative findings at 6 months, we believe there could still be a role for middle turbinectomy in selected, more severe cases. Revision surgeries and/or patients with ‘floppy’ or polypoid turbinates could still be candidates for a future prospective study looking specifically at this topic.

Analysis of individual criteria of both scores showed an increase in crusting in the first month after surgery with resection. Crusts were predominantly seen at the anterior attachment site of the resected middle turbinate, which can be explained by the increased surface of exposed bone during healing. However, this was a transient effect that disappeared once healing was completed and was not associated with adverse outcomes. This pattern is different from the diffuse ethmoid crusting that can be seen in a pathologic sinus cavity plagued with bacterial proliferation, which has a worse prognostic implication. Finally, the proportion of postoperative synechia was unsurprisingly higher on the preserved side.

Our study has some limitations. Because of its design, surgeons could not be blinded to the treatment, the presence or absence of the middle turbinate being obvious at endoscopic evaluation. Symptomatic evaluation of the participants was not possible because of the absence of available tools evaluating nasal symptoms from each nasal cavity independently. This could have been overcome by randomizing patients instead of nasal cavities, but would have taken at least twice the number of participants. The majority of patients underwent primary surgeries, thus results could have been different if revision-only cases were studied, as suggested by Scangas et al. [21]. Finally, a six-month follow-up period may be short considering the chronic course of CRS. Wu et al. showed a longer time interval between sinus surgeries in patients who had undergone middle turbinectomy than in those who had not, but this was shown to happen 4 to 5 years after the first surgery [9]. Our cohort will be followed to assess revision rates.

## Conclusion

Despite previous evidence of increased delivery of nasal topical medication to the sinus cavities after ESS, our results show no objective endoscopic benefits of routine middle turbinectomy in the context of primary surgeries, at least within the first six postoperative months. Limiting the indications for middle turbinectomy to revision surgeries or cases with already problematic turbinates would be a legitimate research question for future prospective studies.

## Abbreviations

CRS: Chronic rhinosinusitis
ESS: Endoscopic sinus surgery
LKES: Lund-Kennedy endoscopic score
POSE: Peri-operative sinus endoscopy
SD: Standard deviation
